# GABRD as an Emerging Oncogene: Exploring Functions and Therapeutic Implications Across Cancers

**DOI:** 10.3390/life16030468

**Published:** 2026-03-13

**Authors:** Tingru Ji, Fengyu Guo, Huaxue Zhang, You Li, Jieying Yuan, Yixuan Wang, Hao Zhang, Xinyu Wang

**Affiliations:** 1 School of Acupuncture and Moxibustion, Shandong University of Traditional Chinese Medicine, Jinan 250355, China; jitingru_1204@163.com (T.J.); 18763056111@163.com (F.G.); 15650097275@163.com (Y.L.); 13153698931@163.com (J.Y.); 2 School of Pharmacy, Shandong University of Traditional Chinese Medicine, Jinan 250355, China; huaxue03042006@163.com (H.Z.); wangyixuan156363@126.com (Y.W.); 3 Institute for Chinese Medicine and Brain Science, Shandong University of Traditional Chinese Medicine, Jinan 250355, China; 4 Key Laboratory of Traditional Chinese Medicine Classical Theory, Ministry of Education, Shandong University of Traditional Chinese Medicine, Jinan 250355, China; 5 Shandong Key Laboratory of Innovation and Application Research in Basic Theory of Traditional Chinese Medicine, Shandong University of Traditional Chinese Medicine, Jinan 250355, China; 6 Shandong Provincial Engineering Research Center for the Prevention and Treatment of Major Brain Diseases with Traditional Chinese Medicine, Shandong University of Traditional Chinese Medicine, Jinan 250355, China

**Keywords:** γ-aminobutyric acid type A receptor subunit delta (GABRD), cancer, cancer cell proliferation, immune microenvironment, colorectal cancer, colon cancer

## Abstract

The γ-aminobutyric acid type A receptor subunit delta (GABRD) constitutes a critical component of the principal inhibitory neurotransmitter receptors within the brain. Recent investigations have revealed aberrant expression of GABRD across a spectrum of non-neural malignancies, including breast, colorectal, and gastric cancers, wherein it exhibits a multifaceted and paradoxical role in oncogenesis. This review delineates the biological characteristics of GABRD and its involvement in cancer pathophysiology. Specifically, the activation of GABRD is implicated in the initiation of key downstream signaling pathways that facilitate the proliferation, invasion, and metastasis of cancer cells. Additionally, the review examines the interaction between GABRD and the tumor microenvironment. Furthermore, it provides an analysis of the diverse roles and mechanisms attributed to GABRD across various cancer types. In conclusion, this review encapsulates the current advancements in understanding the oncogenic functions of GABRD and deliberates on its potential and challenges as a novel target for therapeutic intervention.

## 1. Introduction

The GABA_A_ receptor is a ligand-gated chloride ion channel that serves as a major inhibitory receptor in the central nervous system (CNS) [1]. Its function is mediated by the binding of the inhibitory neurotransmitter γ-aminobutyric acid (GABA) [2]. The composition of various subunits of GABAA receptors determines the receptor’s diverse functional and pharmacological properties. Typically, G_A_BA_A_ receptors are composed of a pentameric assembly of subunits, selected from 19 known isoforms, including GABRA6, GABRB1, GABRG3, GABRD, GABRE, GABRP and GABRQ [3,4]. The δ subunit of the γ-aminobutyric acid A receptor is encoded by the GABRD gene, located on chromosome 1 in humans [2]. According to records from the UniProt database, the GABRD gene consists of approximately 10,000 base pairs and encodes around 452 amino acids.

*GABRD* encodes the δ subunit of the G_A_BAA receptor, which mediates tonic inhibitory currents in the central nervous system [5]. Through its interaction with GABA, GABRD plays an essential role in maintaining neuronal inhibition; mutations or impairments in this gene can disrupt inhibitory signaling, leading to neuronal hyperexcitability and increased susceptibility to seizures [6,7,8]. Beyond its function in neurotransmission, GABRD serves as a biomarker of neuronal exosomes and has been implicated in the diagnosis of Alzheimer’s disease (AD) [9,10]. Its high affinity for neuroactive steroids further underscores its role in regulating neuronal excitability and network stability [11,12], with dysregulation of tonic currents frequently observed in neurological disorders such as epilepsy and anxiety [13].

In recent years, the understanding of GABRD has expanded beyond neurology, with accumulating evidence positioning it as an emerging oncogenic driver across multiple cancer types [14]. GABRD is frequently overexpressed in various solid tumors, and its up-regulation correlates with aggressive clinicopathological features, including advanced tumor stage, higher histological grade, and poor overall survival [14,15]. In neuro-oncology, gliomas—especially glioblastomas—exhibit marked up-regulation of GABRD [16].

**Figure 1:**
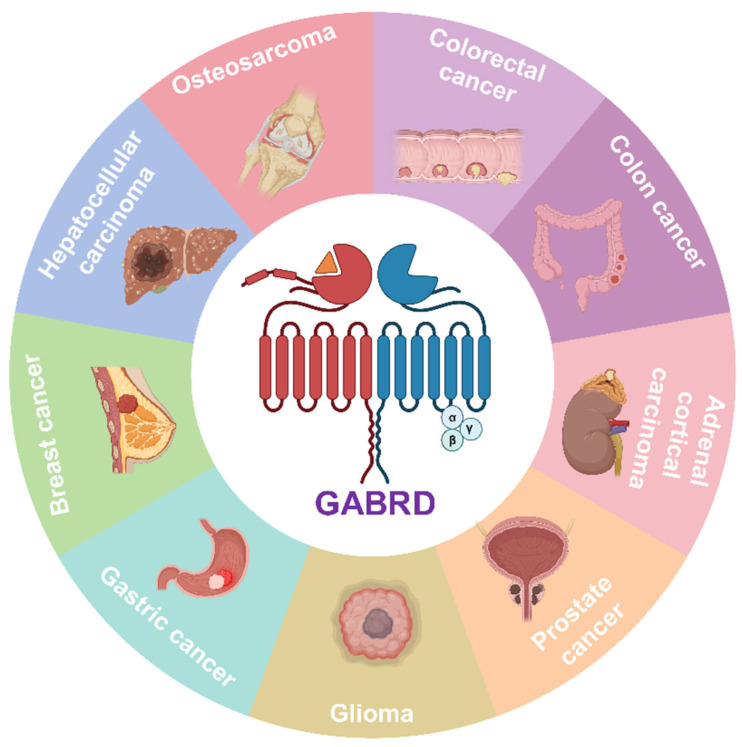
The relationship between GABRD and cancer. Schematic overview illustrating the emerging oncogenic role of GABRD across multiple cancer types. GABRD is frequently overexpressed in various malignancies (e.g., colorectal, breast, gastric, prostate cancer) and its upregulation correlates with poor prognosis, advanced tumor stage, and increased metastatic potential. The central panel highlights GABRD as a shared driver in these cancers, while surrounding icons depict affected cancer types and clinical outcomes. This figure establishes the context for subsequent mechanistic exploration.

Given that tumors are subject to neural regulation through neuroendocrine pathways, the immune microenvironment, and central-peripheral circuits [17,18,19], genes encoding nervous system components also play critical roles in cancer [20,21]. Indeed, GABRD has emerged as a cancer-associated gene with context-dependent functions across different tumor types. Accordingly, this review focuses on the role of *GABRD* in cancer regulation, aiming to elucidate the underlying mechanisms by which GABRD contributes to tumor development and progression, and to explore its dual-modality potential for diagnosis and therapeutic intervention in GABRD-driven malignancies. (Figure 1).

## 2. The Roles and Mechanism of GABRD in Cancer Progression

**Figure 2:**
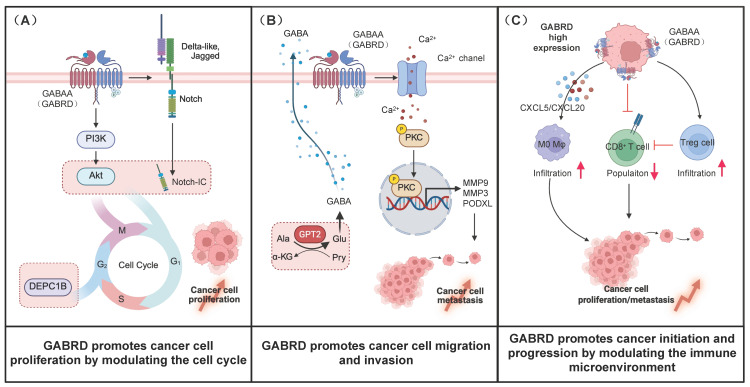
The roles and mechanism of GABRD in cancer progression. (**A**) GABRD promotes cancer cell proliferation by activating PI3K/AKT and Notch signaling pathways, facilitating cell cycle progression (G1/S and G2/M phases). (**B**) GABRD enhances cancer cell migration and invasion via the GPT2–GABA–GABRD–PKC–CREB axis, leading to upregulation of PODXL, MMP3, and MMP9. (**C**) GABRD remodels the tumor immune microenvironment by promoting immunosuppressive cell infiltration (Tregs, M0 macrophages), suppressing CD8^+^ T cell function, and inducing cancer stem cell transformation via Notch signaling.

GABRD, was served as a critical molecule, which was involved in the initiation, progression, and metastasis of tumors. Its high expression promotes the malignant progression of tumors through various mechanisms. The overexpression of *GABRD* not only facilitates the tumor cells’ proliferation and survival by modulating cell cycle-related pathways [22], but also enhances the invasive characteristics of tumor cells by regulating mechanisms such as epithelial–mesenchymal transition (EMT), angiogenesis, and the Hedgehog signaling pathway [14,23]. Furthermore, as a member of the GABAA receptor family, GABRD activates the GABA-PKC-CREB signaling pathway, which upregulates the expression of genes associated with migration and invasion, thereby augmenting the migratory and invasive abilities of tumor cells [24]. Additionally, GABRD contributed to tumor immune escape by modulating immune cell infiltration within the tumor microenvironment. However, the inconsistency in *GABRD* expression levels varies in different types of tumors and plays a different role in each case. Collectively, these findings underscore the crucial roles of GABRD in tumor proliferation, invasion, metastasis, and the regulation of the immune microenvironment (Figure 2)

### 2.1. GABRD Promotes Cancer Cell Proliferation by Modulating the Cell Cycle

The hallmark of cancer is the disruption of normal cell cycle regulation [25]. Irregular cyclin expression disrupts the cell cycle, ultimately contributing to cancer development. Thus, the progression of cancer is closely related to the cell cycle. PI3K-AKT pathway is a major intracellular signaling pathway, and is also a key signal in regulating the cell cycle and cell proliferation in cancer. Notch signaling has been found to regulate cell cycle progression and subsequent cell proliferation through multiple mechanisms [26,27,28]. A study found that co-expressed genes of *GABRD* were significantly enriched in cell cycle-related pathways, including the PI3K-Akt and Notch signaling pathways, and played a role in regulating the G1/S transition of the mitotic cell cycle [22]. In addition, studies suggested that DEPDC1B is closely associated with the G2/M phase of the cell cycle by modulating focal adhesion dynamics [29,30]. Research on esophageal squamous cell carcinoma (ECSS) has shown that GABRD collaborates with DEPDC1B to regulate cancer progression through the PI3K/AKT/mTOR signaling pathway [28,31]. It is tempting to speculate that GABRD might also be involved in cell cycle regulation in ESCC, particularly at the G2/M transition. However, this hypothesis requires direct experimental validation. Moreover, in breast cancer (BC) studies, *GABRD* knockdown induced a significant G2/M phase block in breast cancer cells, thereby suppressing cell [32]. Collectively, these findings demonstrated that GABRD drives cell cycle progression, plays a crucial role in cancer cell cycle regulation, and provides a potential therapeutic target for cancer treatment by regulating the cell cycle to promote proliferation.

### 2.2. GABRD Promotes Cancer Cell Migration and Invasion

Much evidence has proved that *GABRD* is an oncogene, mainly manifested in its ability to facilitate the migration and metastasis of cancer cells. GSEA analysis revealed that the high expression of *GABRD* was positively correlated with key oncogenic events such as EMT, angiogenesis, and the Hedgehog signaling pathway, and in vitro experiments demonstrated that GABRD promoted cancer cell migration, thereby potentially enhancing their invasive capacity [14,23]. A study on gastric cancer revealed that high expression of *GABRD* promotes in the gastric cancer cell lines proliferation migration and invasion, ultimately accelerating the progression of the cancer [33]. Furthermore, glutamine metabolism and GABA signaling pathways also play critical roles in tumor migration and invasion. Increased levels of glutamate pyruvate transaminase 2 (GPT2) catalyze the metabolism of glutamine, leading to increase production of GABA. GABA, through activation of GABA_A_ receptors (including GABRD), opens relevant calcium channels, raising intracellular Ca^2+^ concentrations and subsequently activating the PKC-CREB signaling pathway [24]. It is essential to note that GABRD encodes the δ subunit, which does not function as a standalone receptor but must assemble into a pentameric complex [34]. This activation upregulates the expression of genes such as PODXL, MMP3, and MMP9, thereby enhancing the migration and invasion abilities of breast cancer cells [24,35,36]. As one of the GABA_A_ receptor subunits, GABRD plays a significant role in the GABAA-PKC-CREB signaling pathway, with *GABRD* expression levels being positively correlated with the metastatic potential of breast cancer [24]. In short, high expression of *GABRD* contributed to the progression of various types of cancer by promoting tumor cell migration and metastasis.

### 2.3. GABRD Promotes Cancer Initiation and Progression by Modulating the Immune Microenvironment

GABRD has a significant connection with immune cell infiltration into the tumor microenvironment (TME). As essential constituents of TME, immune cells perform crucial functions in the process of immune surveillance [37]. Tregs primarily exert immunosuppressive effects, inhibiting antitumor immune responses, while M0 macrophages are also closely associated with tumor growth and metastasis [38]. Experimental evidence indicated that *GABRD* expression levels exhibited a significant positive correlation with the abundance of regulatory T cells (Tregs) and M0 macrophages. High expression of *GABRD* is also correlated with a reduction in CD8^+^ T cells, follicular helper T cells, M1 macrophages, activated dendritic cells, and eosinophils. Moreover, the Notch signaling pathway, beyond regulating the cell cycle, is involved in the generation of cancer stem cells(CSC) [39]. Liu et al. reported the co-expressed genes of *GABRD* are highly enriched in the Notch signaling pathway, and the activation of Notch signaling is positively correlated with the generation and maintenance of CSC [22].

## 3. The Mechanism of Action of GABRD in Various Cancers

Given that much evidence has verified that GABRD is an oncogene in most cancers, such as BC, prostate cancer (PCa), and other cancers. However, in glioma, the promoting or inhibiting effect of GABRD is not so clear. Its effect varies depending on the different grades of the glioma. Herein, we will elaborate on the effects and roles of GABRD that contribute to cancer promotion in various types of tumors and the underlying mechanisms (Table 1).

### 3.1. Colon and Rectal Cancer

Colon and rectal cancer are among the most prevalent malignancies worldwide and the most common cancers in the digestive tract, as well as leading causes of cancer-related mortality [50]. Clinical studies have demonstrated that the expression levels of *GABRD* in colon and rectal cancer tissues are significantly higher compared to adjacent normal tissues [14]. This elevated expression is associated with advanced tumor stages and poor patient survival outcomes, suggesting that GABRD could serve as a prognostic biomarker [14,41,43]. Kaplan–Meier analysis further indicates that higher *GABRD* expression is correlated with poor prognosis, increasing risk and offering significant prognostic value, and GABRD is commonly featured in both early screening and prognostic models for colon and rectal cancer [40].

High expression of *GABRD* enriches cancer cells in the G1/S phase of the mitotic cell cycle, promoting cancer cell proliferation by enhancing DNA repair mechanisms [14,22]. Furthermore, *GABRD* co-expression genes are enriched in the Notch signaling pathway, suggesting that GABRD may promote tumor progression by activating Notch signaling to drive cancer stem cell transformation [22]. Furthermore, GABRD’s involvement in key oncogenic pathways, such as epithelial–mesenchymal transition (EMT), angiogenesis, and hedgehog signaling, underscores its potential role in promoting tumor growth and metastasis [14,41,42].

In addition to these pathways, GABRD is involved in modulating the tumor immune microenvironment. Its expression in CRC exhibits a significant positive correlation with the infiltration levels of various immune cells, including B cells, CD4^+^ T cells, CD8^+^ T cells, neutrophils, macrophages, and dendritic cells (DCs) [38]. Specifically in colon adenocarcinoma (COAD), *GABRD* expression is associated with immune cell infiltration, particularly with regulatory T cells (Tregs) and macrophages.

Encouragingly, numerous studies have built upon these findings to develop GABRD-targeting therapies for Colon and rectal cancer, with some demonstrating significant efficacy in experimental settings [51].

### 3.2. Breast Cancer (BC)

Increased glutamine metabolism is a defining characteristic of cancer. GPT2 catalyzes the reversible transamination reaction between alanine and α-ketoglutarate (α-KG), resulting in the production of pyruvate and glutamate [52,53]. This enzymatic reaction plays a pivotal role in cellular glutamine catabolism. Under conditions of metabolic stress, GPT2 expression is significantly upregulated in various tumor cell types, including breast carcinomas [35,36]. The overexpression of GPT2 leads to the activation of GABA_A_ receptors by increasing intracellular GABA levels. Activation of GABA_A_ receptors, in turn, induces an increase in intracellular Ca^2+^ concentration through the opening of calcium channels in the cell membrane, subsequently triggering the PKC-CREB signaling pathway. This activation upregulates the expression of the transmembrane protein PODXL and the extracellularly secreted proteases MMP3 and MMP9, thereby enhancing the migration and invasion abilities of breast cancer cells [24]. Additionally, the expression of the GABRD gene is closely associated with cell cycle regulation, including negative modulation of cell cycle progression and mitotic processes. Research has demonstrated that the deletion of GABRD results in cell cycle arrest in the G2/M phase in breast cancer cells, effectively halting cell division. Conversely, enhanced cell division accelerates tumor progression and contributes to the development of breast cancer [32].

### 3.3. Glioma

Glioma is the most common malignant tumor of neuroepithelial tissue. According to the degree of malignancy, glioma is classified into four grades [18]. Gliomas are classified into low-grade gliomas (WHO grades I–II; LGGs) and high-grade gliomas (WHO grades III-IV; HGGs). Low-grade gliomas are characterized by well-differentiated cellular features [19]. Specifically, diffuse astrocytoma is divided into two subgroups: IDH wild-type (WT) and IDH-mutant (MT).

Synapse and Synapse-associated proteins (Saps) play crucial roles in the pathogenesis of various brain tumors [54,55,56]. Lin et al. [44] identified a significant association between four SAPs—glutamate ionotropic receptor kainate type subunit 2 (GRIK2), GABRD, glutamate ionotropic receptor type subunit 2 (GRID2), and activity-regulated cytoskeleton-associated protein (ARC)—and the progression and prognosis of LGG. Their findings, derived from Western blot and quantitative real-time PCR (qRT-PCR) analyses, demonstrated that the expression levels of GRIK2 and GRID2 were elevated in glioma tissues compared to normal brain tissues, whereas GABRD expression was downregulated. These results suggest that these four SAPs are involved in the formation of glutamate synapses within LGG. Furthermore, studies on IDH-WT-LGG have revealed that *GABRD* expression is reduced and have established a weak negative correlation between GABRD and CCL2/EGF, as well as a moderate negative correlation with CSF1 [45]. Experimental data indicate that maintaining *GABRD* expression may inhibit tumor progression and improve patient outcomes by reducing tumor-associated macrophage infiltration (TIM). The observed downregulation of *GABRD* expression in glioma tissues further supports the involvement of these SAPs in the formation of glutamate synapses [45].

HGGs, including glioblastoma multiforme (GBM), are classified as poorly differentiated gliomas. These tumors are recognized as highly malignant neoplasms, often characterized by an exceptionally poor prognosis. GBM, the most common subtype of glioma, is notably associated with a 5-year survival rate of approximately 5.6% [57]. Yang et al. [46] found that several genes including *GABRD* are differentially expressed in GBM and LGG, that is, unlike *GABRD* in LGG, elevated expression of *GABRD* has a negative impact on the prognosis of GBM patients. This experiment also speculated that differentially expressed genes (DEGs) such as *GABRD* were mainly enriched in the ion signaling mutual pathway to affect the occurrence and development of GBM [46].

### 3.4. Other Cancers

In addition to the multiple mechanisms of action of GABRD in the aforementioned cancers, its role in other cancers has also garnered widespread attention. A study [47] by highlights the role of *GABRD* in PCa, demonstrating that its silencing in endothelial cells can attenuate tube formation and inhibit PCa cell proliferation, suggesting a potential therapeutic target for managing PCa recurrence [47]. In addition to its role in PCa, *GABRD* has been identified as a novel oncogene in gastric cancer, where its knockdown induces apoptosis and cell cycle arrest, thereby repressing proliferation and migration [15]. Arjun et al. discovered the specific expression of *GABRD* in stage IV hepatocellular carcinoma (HCC), suggesting that its upregulation might play a critical role in the proliferation and independent differentiation of HCC cells [49,58]. Another study indicated that *GABRD* knockdown reduced the phosphorylation levels of AKT and mTOR, which suggests that *GABRD* might regulate the PI3K/AKT/mTOR oncogenic signaling pathway, thereby influencing cancer initiation and progression [28]. Knott et al. [23] found that *GABRD* transcripts were detectable in almost all adrenal ACC tumor cells, and its expression levels were negatively correlated with ABAT transcripts, implying that GABRD may contribute to poor prognosis in ACC patients by reducing the degradation capacity of GABA [23]. Additionally, *GABRD* expression was significantly upregulated in osteosarcoma (OS) cell lines, suggesting that GABRD might play a role in the initiation and progression of OS [48]. Further studies indicate that GABRD may promote cancer progression by affecting the function of immune cells within the TME or by regulating processes such as tumor cell proliferation and migration [48,59,60].

## 4. Conclusions and Perspectives

Current research delineates a predominantly pro-tumorigenic role for GABRD across multiple cancer types. However, it is important to note that this role is not yet fully established for all malignancies. In low-grade gliomas, GABRD appears to function as a tumor suppressor rather than an oncogene, while its role in other cancers such as osteosarcoma and hepatocarcinoma remains to be elucidated, as summarized in Table 1. As a cancer-promoting gene, the mechanisms whereby GABRD’s role in tumors mainly consists of three aspects. Primarily, high expression of *GABRD* can promote the proliferation of some types of tumor cells. Such as, elevated *GABRD* expression has been shown to promote the enrichment of Colon cancer cells in the G1/S phas, thereby facilitating cell proliferation [22]. The deletion of GABRD can block the progression of BC cells through the G2/M phase of mitosis, inhibiting their proliferation [32]. Second, *GABRD* expression is significantly positively correlated with the infiltration of various immune cell types. GABRD exhibits significant correlations with immune cells, such as regulatory T cells, M0 macrophages, and CD8^+^ T cells, suggesting that GABRD may contribute to the formation of an immunosuppressive tumor microenvironment, thereby promoting tumor initiation and progression. Additionally, overexpression of *GABRD* has been found to induce the transformation of cancer cells into cancer stem cells via the activation of the Notch signaling pathway, thus accelerating the progression of CRC [22,39]. Third, GABRD modulates the tumor immune microenvironment by influencing immune cell infiltration. Increased activation of GABA_A_ receptors in BC cells induces the PKC-CREB signaling pathway, leading to the upregulation of intracellular PODXL, MMP3, and MMP9, which subsequently promotes BC tumor metastasis [24,35,36]. Additionally, GABRD plays a pivotal role in the initiation and progression of ECSS through the regulation of the PI3K/AKT/mTOR pro-cancer signaling pathway [28]. GABRD is implicated in integrin binding and can induce apoptosis by modulating the viability and cell adhesion of CC cells. Silencing *GABRD* expression results in the inhibition of migration and invasion of HUVECs and suppresses the proliferation of PC-3 and 22Rv1 cells, leading to a reduction in tube formation ability and inhibiting the proliferation and migration of PCA cells [47]. However, GABRD is an inhibitor in LGG, and is involved in the formation of glutamatergic synapses and LGG synapses and inhibit tumor progression and improve patient prognosis by reducing TIM infiltration [45]. In HGGs, high expression of *GABRD* has a negative impact on the prognosis of GBM patients [46]. In summary, GABRD emerges as a molecule of significant importance in oncology, exhibiting a dual role that can either promote or suppress tumor progression depending on the cancer type.

While numerous studies have demonstrated a robust association between GABRD and various cancer types, further research is imperative to elucidate its precise role and the underlying mechanisms in these malignancies. Specifically, the upstream and downstream signaling networks of *GABRD* across different cancer types require further elucidation. Additionally, its function within the tumor microenvironment and its interactions with the immune system represent emerging areas of inquiry. Mechanistically, GABRD engages in crosstalk with epidermal growth factor receptor (EGFR) and other receptor tyrosine kinases (RTKs), thereby amplifying mitogenic signaling and promoting tumor proliferation. Epigenetic alterations, particularly hypomethylation of the GABRD promoter, often drive this aberrant expression. Moreover, the enrichment of GABRD in circulating tumor DNA (ctDNA) and tumor-derived exosomes highlights its potential as a non-invasive biomarker for liquid biopsy and real-time disease monitoring. Beyond basic mechanisms, the clinical utility of GABRD in surgical and medical oncology warrants deeper exploration. Specifically, future investigations should determine whether *GABRD* expression fluctuates during neoadjuvant or adjuvant therapies, potentially serving as a dynamic indicator of treatment sensitivity or resistance. Moreover, given the stark contrast in *GABRD* expression between malignant and adjacent normal tissues, it could theoretically be evaluated as a molecular tool for R1 resection assessment to improve surgical precision. Most importantly, the established correlation between high *GABRD* levels and poor postoperative outcomes supports its development as a prognostic biomarker for real-time disease monitoring and early detection of recurrence during long-term follow-up. Consequently, continued investigation is crucial to establish a comprehensive foundation for the potential therapeutic application of GABRD inhibitors or activators in cancer treatment.

In summary, GABRD has emerged as a potential anti-cancer target, attracting considerable attention in recent years. Although traditionally recognized for its role in inhibiting neural transmission in the brain, studies have indicated its aberrant expression in various cancers, where it contributes to tumor progression through unique mechanisms. Therefore, targeting GABRD may provide a novel and alternative anti-cancer strategy distinct from conventional therapies.

## Figures and Tables

**Table 1:** Roles and action mechanisms of GABRD in cancer.

Cancer Types	Role of GABRD	Action Mechanisms of GABRD	Species/Research Subjects	Reference
Colon and Rectal Cancer	Oncogenic	Promotes G1/S phase transition and DNA repair, thereby enhancing proliferation. Activates Notch signaling, leading to cancer stem cell transformation. Directly inhibits CD8+ T cells via GABRD receptor signaling. Promotes EMT, angiogenesis, and Hedgehog pathway.	Human tissues, human cell lines	[14,22,38,40,41,42,43]
Breast cancer	Oncogenic	Activates the PKC-CREB pathway through the GPT2–GABA–GABRD axis, thereby upregulating PODXL, MMP3, and MMP9, which in turn enhances migration and invasion. Regulates the G2/M checkpoint, thereby promoting cell cycle progression.	Human cell lines, xenograft models (mouse)	[24,32]
Low-grade gliomas	Tumor suppressive	Downregulated in LGG; maintains glutamatergic synapse formation. Preserving GABRD expression reduces TIM infiltration, thereby inhibiting progression and improving prognosis.	Human tissues, bioinformatic analysis	[44,45]
Glioblastoma multiforme	Possible oncogenic	Upregulated in GBM; enriched in ion signaling pathways; negative prognostic impact.	Human tissues, bioinformatic analysis	[46]
Prostate cancer	Oncogenic	Silencing GABRD in endothelial cells attenuates tube formation and inhibits PCa cell (PC-3, 22Rv1) proliferation	Human cell lines, endothelial cells	[47]
Gastric cancer	Oncogenic	High expression promotes proliferation, migration, and invasion in gastric cancer cell lines	Human cell lines	[33]
Osteosarcoma	Oncogenic	Unclear	Human cell lines	[48]
Hepatocellular carcinoma	Oncogenic	Unclear	Human tissues, bioinformatic analysis	[49]
Adrenocortical carcinoma	Oncogenic	GABRD expression in ACC, inversely correlated with ABAT, may impair GABA degradation and drive poor prognosis.	Human tissues, bioinformatic analysis	[23]

## Data Availability

No new data were created or analyzed in this study. Data sharing is not applicable to this article.

## Abbreviations

ACC, adrenal cortical carcinoma; AD, Alzheimer’s disease; ARC, activity-regulated cytoskeleton-associated protein; BC, Breast cancer; CNS, central nervous system; COAD, colon adenocarcinoma; CRC, colorectal cancer; CSC, cancer stem cells; DEGs, differentially expressed genes; EMT, epithelial–mesenchymal transition; ECSS, esophageal squamous cell carcinoma; GABA, γ-aminobutyric acid; GABRD, γ-aminobutyric acid type A receptor subunit delta; GBM, glioblastoma multiforme; GRID2, glutamate ionotropic receptor type subunit 2; GRIK2, glutamate ionotropic receptor kainate type subunit 2; GSEA, Gene Set Enrichment Analysis; GPT2, Glutamic pyruvate transaminase 2; HCC, hepatocellular carcinoma; HGGs, high-grade gliomas; HUVEGs, human umbilical vein endothelial cells; LGGs, low-grade gliomas; MT, mutant; OS, osteosarcoma; PCa, prostate cancer; qRT-PCR, quantitative real-time PCR; Saps, Synapse-associated proteins; TIM, tumor-associated macrophage infiltration; TME, tumor microenvironment; WT, wild-type; GRIK2, glutamate ionotropic receptor kainate type subunit 2; GRID2, glutamate ionotropic receptor type subunit 2; ARC, activity-regulated cytoskeleton-associated protein; α-KG, α-ketoglutarate.
